# No differences between 4‐strand semitendinosus or semitendinosus/gracilis grafts in revision rates, knee stability and patient‐reported outcomes after primary anterior cruciate ligament reconstruction

**DOI:** 10.1002/jeo2.70232

**Published:** 2025-04-04

**Authors:** Torsten Grønbech Nielsen, Martin Lind

**Affiliations:** ^1^ Division of Sports Traumatology, Department of Orthopedic Aarhus University Hospital Aarhus Denmark; ^2^ Department of Physiotherapy and Occupational Therapy Aarhus University Hospital Aarhus Denmark

**Keywords:** anterior cruciate ligaments, autografts, hamstring muscles, knee injury, treatment outcome

## Abstract

**Purpose:**

The purpose of this study was to compare revision rates, knee stability and patient‐reported outcomes in a national cohort of anterior cruciate ligament (ACL) reconstructed patients using either the semitendinosus/gracilis (ST/G) or the 4‐strand semitendinosus (4‐ST) grafts.

**Methods:**

ACL reconstructed patients operated from 2015 to 2021 who met the following criteria: minimum 2‐year follow‐up and isolated ACL with either ST/G or 4‐ST grafts. The primary outcome was ACL revision surgery assessed at 2‐year follow‐up. Secondary outcomes were knee laxity (side‐to‐side difference) and pivot shift (rotational stability difference—Grade 0 or Grade 1–3), and patient‐reported outcomes; Knee Osteoarthritis and Outcome Score (KOOS) subscales and Tegner activity scale assessed at 1‐year follow‐up.

**Results:**

A total of 6750 ST/G and 1321 4‐ST patients were included in the study. There was no statistical difference in 2‐year revision rates between the groups (ST/G; 1.73 (95% confidence interval [CI] 1.44–2.07), 4‐ST; 1.40 (95%CI 0.88–2.21)). A small significant but not clinically relevant difference was seen in knee laxity (1.27 mm vs. 1.13 mm), but no other significant differences were seen in pivot shift or patient‐reported outcomes at one year. Both groups showed significant improvement from baseline to 1 year.

**Conclusion:**

The present study found no difference between ST/G or 4‐ST in ACL reconstruction patients regarding revision rates, knee laxity and patient‐reported outcomes.

**Level of Evidence:**

Level III.

**Abbreviations**
4‐ST: four‐strand semitendinosus graft
ACL: anterior cruciate ligament
Adj.: adjusted
ADL: activity of daily living
CI: confidence interval
DKLR: Danish Knee Ligament Reconstruction Registry
HR: hazard ratio
ICRS: International Cartilage Regeneration & Joint Preservation Society
KOOS: Knee Injury and Osteoarthritis Outcome Score
mm.: millimetre
n: numbers
PROMS: Patient‐Reported Outcome
QOL: quality of life
SD: standard deviation
ST/G: semitendinosus/gracilis graft

## BACKGROUND

The optimal choice of graft for anterior cruciate ligament (ACL) reconstruction remains uncertain. Various graft types such as hamstring tendon, patellar tendon and quadriceps tendon are the most used [14, 15].

The most commonly used hamstring graft is the double‐stranded semitendinosus and gracilis (ST/G), which has shown low revision rates, good sagittal stability and excellent patient‐reported outcomes (PROMS) [21]. A still debated disadvantage of using the ST/G is increased revision rates compared to patellar tendon grafts [6], rotational instability, the difficulty in achieving the optimal graft diameter and donor site morbidity in the form of reduced hamstring strength [3, 18].

The four‐strand semitendinosus graft (4‐ST) has shown the same good results in terms of knee stability and PROMS compared to ST/G [21, 27]. The use of 4‐ST makes it easier to achieve an optimal graft diameter if the graft length is long enough to secure a 4‐strand graft [18]. The technique involves only the semitendinosus muscle leaving the gracilis untouched. A disadvantage of using a 4‐ST is the possibility of harvesting a tendon that is too short, making it impossible to achieve a secure fixation in the tibia and femur. If the surgeon harvests too short a semitendinosus tendon, an additional gracilis tendon can be harvested and combined.

A systematic review comparing ST/G and 4‐ST found no statistical difference in sagittal knee laxity and PROMS but found small statistical differences in knee flexion muscle strength. These were not considered to be clinically relevant [26].

Only one study of approximately 7000 patients from a single centre has compared two‐year revision rates and found 1.7% and 2.1% for ST/G and 4‐ST, respectively [4]. The present study will provide new evidence on revision rates, knee stability and subjective outcomes based on a national cohort.

The primary purpose of this study was to compare revision rates after ACL reconstruction using either ST/G or 4‐ST grafts at 2 years' follow‐up. It was hypothesised that 4‐ST revision rates would be twice as high as STG revision rates at 2 years. Second, to compare knee laxity and PROMS at 1 year.

## MATERIAL AND METHODS

As the study consists of registry data only, local ethics committee approval is not required and therefore, consent was not achieved. The study has been registered according with the Danish Data Protection Agency (1‐16‐02‐433‐24).

The study data were extracted from the Danish Knee Ligament Reconstruction Registry (DKLR). The DKLR was established in 2005 and is a national registry that includes all ACL reconstructed patients. Registration of ACL reconstructed patients is mandatory for all surgeons. The prospective data collected included pre‐, peri‐ and post‐operative data. Pre‐operative data included age, sex, injury date, mechanism of injury, knee laxity and PROMS (Knee Injury and Osteoarthritis Outcome Score [KOOS] and the Tegner Activity Score [Tegner]). Peri‐operative data included cartilage damage/treatment, meniscus treatment, graft selection, graft fixation and registration of surgery date. Post‐operative data collected at one year included assessment of knee laxity and PROMS (KOOS and Tegner). The PROMS were web‐based and were completed by patients both preoperatively and postoperatively. ACL revision surgeries were linked to patients by their social security number and were recorded in the period after the primary ACL reconstruction.

### Study population

The population consisted of patients undergoing primary isolated ACL reconstruction who underwent surgery between 2015 and 2021. Inclusion criteria were age' >18 years, registered graft choice (ST/G or 4‐ST), primary isolated ACL reconstruction and at least 2 years of follow‐up.

A total of 42,347 ACL reconstructed patients were extracted from the DKLR. 34,276 patients did not meet the inclusion criteria and were excluded (not within the inclusion period: 27,475 patients, age < 18 years: 2418 patients, ACL revision: 1022 patients, multiligament: 147 patients, graft other than ST/G and 4‐ST: 3214 patients). Finally, 8071 ACL reconstructed patients were included in the total study cohort, of whom 6750 had an ST/G graft and 1321 had a 4‐ST graft (Table 1).

**Table 1:** Baseline characteristics with comparison of the two cohorts (ST/G and 4‐ST).

	ST/G	4‐ST	*p* value
Patients (*n*)	6750	1321	
Age, mean (SD)	30.0 (9.6)	30.6 (10.0)	0.03
Sex			
‐ Male, *n* (%)	4163 (61.7)	880 (66.6)	<0.001
‐ Female, *n* (%)	2587 (38.3)	441 (33.4)	
Knee Laxity (mm), mean (95%CI)	4.80 (4.75–4.85)	4.59 (4.50–4.69)	<0.001
Pivot shift			
‐ Grade 0, *n* (%)	779 (12.1)	91 (7.0)	<0.001
‐ Grade 1, *n* (%)	2469 (38.5)	478 (36.7)	
‐ Grade 2, *n* (%)	2678 (41.8)	698 (53.6)	
‐ Grade 3, *n* (%)	488 (7.6)	36 (2.8)	
Graft			
‐ Size (mm), mean (95%CI)	8.22 (8.20–8.24)	8.73 (8.69–8.76)	<0.001
‐ <8 mm, *n* (%)	1405 (20.9)	71 (5.4)	<0.001
‐ ≥8 mm, *n* (%)	5319 (79.1)	1247 (94.6)	
Time from injury to surgery (days), mean (SD)	569 (1082)	476 (914)	0.004
Injury mechanism			
‐ Sport, *n* (%)	5495 (81.4)	1099 (83.2)	0.07
‐ ADL, *n* (%)	687 (10.2)	113 (8.6)	
‐ Traffic, *n* (%)	166 (2.4)	24 (1.8)	
‐ Work, *n* (%)	160 (2.4)	42 (3.2)	
‐ Other, *n* (%)	342 (3.6)	43 (3.2)	
Cartilage damage			
‐ None	5304 (78,6)	987 (74.7)	0.001
‐ ICRS 1–2, *n* (%)	1115 (16.5)	273 (20.7)	
‐ ICRS 3‐4, *n* (%)	331 (4.9)	61 (4.6)	
Meniscus treatment			
‐ None	3751 (55.6)	691 (52.3)	<0.001
‐ Repair, *n* (%)	1257 (18.6)	313 (23.7)	
‐ Resection, *n* (%)	1742 (25.8)	317 (24.0)	
PROMS, *n* (%)	2284 (34%)	376 (28%)	
‐ KOOS‐Pain, median (25th–75th)	72 (58–83)	74 (58–86)	0.12
‐ KOOS‐Symptoms, median (25th–75th)	71 (57–82)	73 (61–86)	0.04
‐ KOOS‐ADL, median (25th–75th)	82 (68–91)	82 (68–93)	0.41
‐ KOOS‐Sport, median (25th–75th)	30 (15–55)	35 (15–55)	0.16
‐ KOOS‐QOL, median (25th–75th)	38 (25–50)	38 (31–50)	0.06
‐ Tegner, median (25th–75th)	3 (2–4)	3 (2–4)	0.82

Abbreviations: 4‐ST, 4‐strand semitendinosus; ADL, activity of daily living; ICRS, International Cartilage Regeneration & Joint Preservation Society; KOOS, Knee Injury and Osteoarthritis Outcome Score; mm, millimetres; *n*, numbers; PROMS, patient reported outcomes; QOL, quality of life; SD, standard deviation; ST/G, semitendinosus/gracilis.

Statistically significant differences were found between the baseline characteristics of the two groups with respect to age, sex, knee laxity, graft size, time from injury to surgery, cartilage injury and meniscal treatment. There were no differences in mechanism of injury or PROMS between the two cohorts (Table 1).

### Outcomes measures

The primary outcome was graft failure, defined as an ACL revision surgery. Failure rates were calculated for each graft type and the ratio of ST/G to 4‐ST was calculated, adjusted for potential confounders. All ACL revision procedures were recorded between 2015 and 2023.

Secondary outcomes included both sagittal and rotational knee laxity and subjective knee function.

Sagittal knee laxity was measured using maximum manual force with a KT‐1000 arthrometer or a Rolimeter [1, 5]. The side‐to‐side difference between the healthy and injured knee was measured and reported in millimetres.

The pivot shift test was used to measure rotational knee laxity. The difference between the healthy and injured knee was graded using a 4‐point Likert scale. The grading was: Grade 0 (normal), Grade 1 (glide), Grade 2 (clunk) and Grade 3 (locked subluxation) [7]. Positive pivot shifts were considered as Grades 1–3.

Subjective knee function was assessed using the PROMS, including the KOOS and Tegner [2, 24].

The KOOS consists of five domains (symptoms, pain, activity of daily living [ADL], sport and quality of life [QOL]), all ranging from 0 to 100, with 100 being best.

The Tegner ranged from 0 to 10, with a score of 10 being elite level. Secondary outcomes were measured and collected preoperatively and at 1‐year follow‐up.

### Statistical analysis

Continuous data were tested for normal distribution and were presented as mean with standard deviation (SD), mean with 95% confidence interval (95% CI). Non‐distributed data were presented as medians with 25th and 75th percentiles. Proportions were presented as numbers (*n*) and percentages (%).

Comparisons were made using the student's *t*‐test for normally distributed continuous data (age and knee laxity), Wilcoxon rank sum test for non‐distributed continuous data (PROMS) and Chi^2^ test for proportions (sex, pivot shift, injury mechanism, cartilage damage and meniscal treatment).

The risk of ACL revision was calculated. A power calculation for this study to minimise type 2 errors was performed using a significance level (*α*) of 0.05 and two‐year revision rates of 2% in the STG group and 4% in the 4‐ST group to assess the difference in proportions between the two groups. With level (*α*), revision rates of 2% in 6493 patients and 4% in 1267 patients, the power was estimated at 97.1%. This is above the 80% power level that is considered good for most trials. The trial is therefore well powered to detect a difference, if one exists.

The adjusted hazard ratio (HR) was used to compare the risk of an ACL revision between 4‐ST and ST/G surgery. Potential confounders (age, sex, knee laxity, pivot shift, graft size, cartilage damage (ICRS: International Cartilage Regeneration & Joint Preservation Society), and meniscal treatment) were adjusted for in the HR calculation. Two‐year cumulative ACL revision failure rates were calculated using the Kaplan–Meier method. *p*‐Values < 0.05 were considered statistically significant. Data analyses were performed with Stata 18.0 (Stata Corp).

## RESULTS

**Figure 1:**
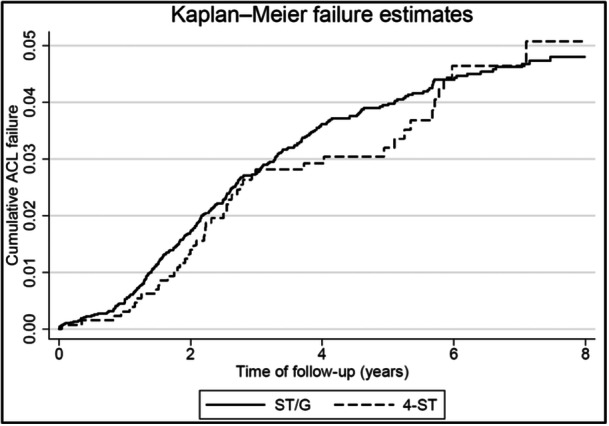
Kaplan–Meier revision estimates. 4‐ST, 4‐strand semitendinosus; ACL, anterior cruciate ligament; ST/G, semitendinosus/gracilis.

No difference was found in revision rates between ST/G (1.73 [95%CI 1.44–2.07]) and 4‐ST (1.40 [95%CI 0.88–2.21]) at 2 years or after adjustment for potential confounders (adj.HR 0.99 [0.70–1.38]) (Table 2) and (Figure 1).

**Table 2:** Two‐years ACL revision rates for ST/G graft and 4‐ST graft.

	ACL‐revision rate (95%CI)	Adj.HR (95%CI)	*p* value
ST/G graft (*n* = 6493)	1.73 (1.44–2.07)	*Reference*	
4‐ST graft (*n* = 1267)	1.40 (0.88–2.21)	0.99 (0.70–1.38)	0.94

*Note*: HR: Adjusted for age, sex, knee laxity, pivot shift, graft size, cartilage damage and meniscus treatment.

Abbreviations: 4‐ST, 4‐strand semitendinosus; ACL, anterior cruciate ligament; Adj., adjusted; CI, confidence interval; HR, hazard ratio; *n*, numbers; ST/G, semitendinosus/gracilis.

A significant difference (*p* = 0.004) in sagittal stability was found between ST/G (1.27 mm) and 4‐ST (1.13 mm) at 1 year. No difference in rotational stability was found between the two graft types in patients with negative and positive pivot shift at one year (Table 3). No statistical differences were found for KOOS and Tegner at 1 year (Table 4).

**Table 3:** Postoperative objective knee laxity and pivot shift results after ACL reconstruction at 1‐year.

	ST/G	4‐ST	*p* value
	*n* = 3736 (55%)	*n* = 919 (70%)	
Knee laxity (mm), mean (95%CI)	1.27 (1.23–1.31)	1.13 (1.04–1.22)	0.004
Pivot shift	*n* = 4080 (60%)	*n* = 980 (74%)	
‐ Grade 0, *n* (%)	3484 (85.4)	856 (87.4)	0.12
‐ Grade 1–3, *n* (%)	596 (14.6)	124 (12.6)	

Abbreviations: 4‐ST, 4‐strand semitendinosus; ACL, anterior cruciate ligament; CI, confidence interval; mm,millimetres; *n*, numbers; ST/G, semitendinosus/gracilis.

**Table 4:** Postoperative patient reported outcomes after ACL reconstruction at 1‐year.

	ST/G	4‐ST	*p* value
PROMS, *n* (%)	1779 (26%)	339 (26%)	
‐ KOOS‐pain, median (25th–75th)	86 (72–94)	86 (72–74)	0.96
‐ KOOS‐symptoms, median (25th–75th)	79 (64–89)	75 (60–86)	0.17
‐ KOOS‐ADL, median (25th–75th)	93 (82–97)	93 (84–99)	0.48
‐ KOOS‐sport, median (25th–75th)	65 (40–80)	65 (40–85)	0.30
‐ KOOS‐QOL, median (25th–75th)	56 (44–69)	56 (44–75)	0.58
‐ Tegner, median (25th–75th)	5 (4–6)	5 (4–6)	0.14

Abbreviations: 4‐ST, 4‐strand semitendinosus; ACL, anterior cruciate ligament; ADL, activity of daily living; KOOS, Knee Injury and Osteoarthritis Outcome Score; PROMS, patient reported outcomes; QOL, quality of life; ST/G, semitendinosus/gracilis.

## DISCUSSION

The primary finding of the study was that there was no difference in revision rates between ST/G and 4‐ST at two years. The hypothesis that 4‐ST revision rates would be twice those of ST/G was rejected.

Only one previous study conducted by Cristiani et al. [4] was based on sufficient patient volume to present revision rates for ST/G and 4‐ST revision rates. Compared to the present study, the Christiani study found a similar 2‐year revision rate for ST/G of 1.7%. However, Cristiani et al. found a lower revision rates for 4‐ST at 1.4% and 2.1%, respectively [4]. This small difference is unlikely to be clinically relevant. Compared with other graft types, hamstring grafts have previously been shown to have slightly higher revision rates than patellar tendon grafts [23]. When compared with patellar tendon grafts from Scandinavian registry data, 2‐year revision rates were found to be as low as 1.0% [6], which is comparable to the data from this study.

When comparing revision rates with quadriceps graft revision rates from the DKLR, the revision rates in this study were 1.4% and 1.7%, respectively, which is lower than 2.8% [16]. When comparing the revision rates from the present study with the two‐year revision rates for quadriceps grafts from DKLR, the revision rates in the present study were 1.4%–1.7%, which was lower than the 2.8% found for quadriceps [16]. This higher revision rate for quadriceps grafts may be due to a learning curve, as the quadriceps tendon is still a newer graft type compared to the hamstring grafts, which is also stated in a previous study [17].

There was a significant difference in sagittal knee laxity between the two groups (ST/G 1.27 mm, 4‐ST 1.13 mm) at 1 year follow up. The difference was small and not considered clinically relevant. Sagittal knee laxity in the present study was comparable to previous studies comparing ST/G and 4‐ST. A 2019 meta‐analysis of data from 11 studies comparing ST/G and 4‐ST found no differences in sagittal knee laxity [3]. Knee laxity ranged from 1.1 to 2.2 mm and 1.0 to 2.3 mm in the ST/G and 4‐ST groups, respectively. A recent randomised controlled trial found no difference in sagittal knee laxity between groups 4.5 years after ACL reconstruction (ST/G 0.73 mm vs. 4‐ST 1.40 mm) [12].

The recently published large single centre study by Cristiani et al. [4] found a 1‐year knee laxity of ST/G 2.1 mm versus 4‐ST 1.7 mm after ACL reconstruction in a cohort of approximately 7000 patients. Although there are small differences between this study and the studies presented, these small differences are likely without clinical relevance and as well the sagittal knee laxity is below the 2 mm threshold indicating normal knee stability [8].

There was no significant difference in rotational stability between ST/G and 4‐ST at 1 year follow up. Stability of rotation in terms of pivot shift has previously been reported in studies comparing ST/G and 4‐ST, with no rotational instability estimated at be 76%–98% and 78%–98% for ST/G and 4‐ST, respectively. Variation between studies has been noted, but no studies have shown differences between groups [9, 12, 13, 25, 27].

Subjective clinical outcomes based on the KOOS have previously been presented in comparative studies between ST/G and 4‐ST and found no significant difference between graft types for all subscores, which is comparable to the present study [4, 11, 12, 21]. As KOOS was originally developed for patients with osteoarthritis, only the KOOS‐Sport and KOOS‐QOL are evaluated as valid parameters in ACL reconstructed patients [10]. KOOS‐Sport and KOOS‐QOL are lower in the present study compared to the studies with 3 and 4 years follow‐up [12, 21], but the results are comparable to the study by Cristiani et al. [4] presenting data from a Swedish population comparable to the cohort of this study. The lower KOOS scores in this study compared to studies with longer follow‐up may be a consequence of patients not being allowed to participate in contact sports within the first year. This is due to a general rehabilitation recommendation of not returning to pivoting sports until 12 months after ACL reconstruction due to the increased risk of ACL graft failure.

### Limitations

A limitation of the study is the lack of hamstring muscle strength in both groups, which could influence the difference in harvesting technique. However, muscle strength data are not collected and recorded in the DKLR. Another limitation is the low completeness of the PROMS, which is known to be a problem in registry‐based studies [19, 20]. This low completeness may not affect the results, as a previous study found no difference in outcomes between responders and non‐responders [22].

Another limitation is the lack of corrections for variations in the graft fixation implants used. As numerous combinations of graft fixation implants were used in this national cohort study, it is not possible to include or correct for this factor in the outcome analyses.

## CONCLUSION

The present study found no difference between ST/G or 4‐ST in ACL reconstruction patients regarding revision rates, knee laxity and patient‐reported outcomes.

## AUTHOR CONTRIBUTIONS

All authors contributed to the study conception and design. Data analysis were performed by Torsten Grønbech Nielsen. Analysis and interpretation of the results was done by both authors. Martin Lind was a major contributor in writing the manuscript. Both authors read and approved the final manuscript.

## CONFLICT OF INTEREST STATEMENT

The authors declare no conflicts of interest.

## ETHICS STATEMENT

As this study is based on registry data, approvement from the local ethics committee is not needed. Due to data was collected from a national registry and an internal database informed consent was not required.

## CLINICAL TRIAL REGISTRATION

As the study is not a clinical trial it is not registered at the www.clinicaltrials.gov.

## Data Availability

Data are available on request.
